# Supplementary material for: Nutritional Value of Banded Cricket and Mealworm Larvae
Source: Foods. 2023 Nov 20;12(22):4174. doi: 10.3390/foods12224174 (PMC10670232; doi:10.3390/foods12224174)
Supplement: Supplementary file 1 [file foods-12-04174-s001.zip › foods-2689706-supplementary.pdf]

**Table S1.** Mineral composition of certified reference material (skimmed milk powder (ERM®-BD151)).

| Item | Measured value | Certified value [37] |
|---|---|---|
| Macroelements (g/100 g) | | |
| P | 1.0525±0.0432 | 1.0952±0.0462 |
| K | 0.1245±0.0019 | 1.7009±0.0583 |
| Ca | 1.4292±0.0846 | 1.3888±0.0595 |
| Na | 0.4095±0.0078 | 0.4195±0.0140 |
| Mg | 0.1225±0.0482 | 0.1264±0.0047 |
| Microelements (mg/100 g) | | |
| Zn | 4.6810±0.0502 | 4.4870±0.2120 |
| Fe | 5.1300±0.0218 | 5.2730±0.2420 |
| Cu | 0.4785±0.0043 | 0.4999±0.0270 |
| Mo | 0.0001±0.0000 | N |
| Mn | 0.0268±0.0005 | 0.0292±0.0030 |
| Heavy metals (mg/100 g) | | |
| Pb | 0.0215±0.0071 | 0.0207±0.0010 |
| Cd | 0.0095±0.0001 | 0.0106±0.0009 |
| Ni | 0.0003±0.0000 | N |
| Co | 0.0004±0.0000 | 0.0006±0.0002 |
| Cr | 0.0001±0.0000 | N |

N, not included in the certified material
